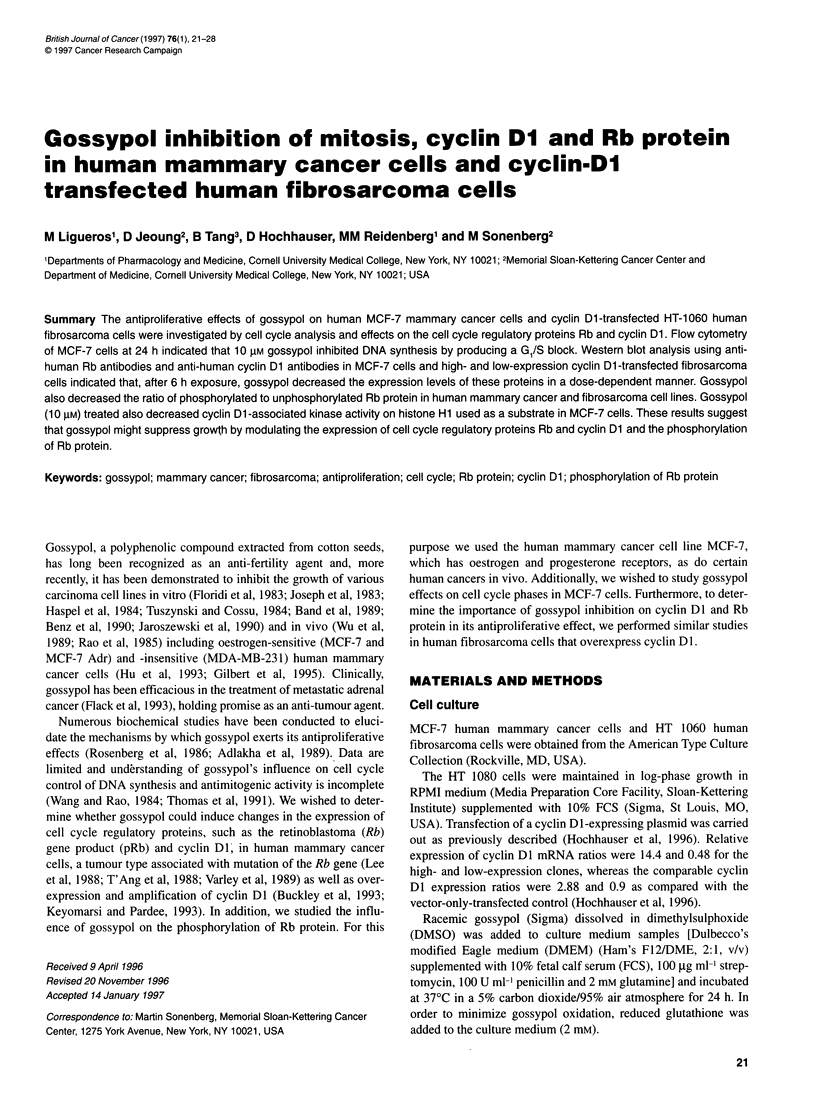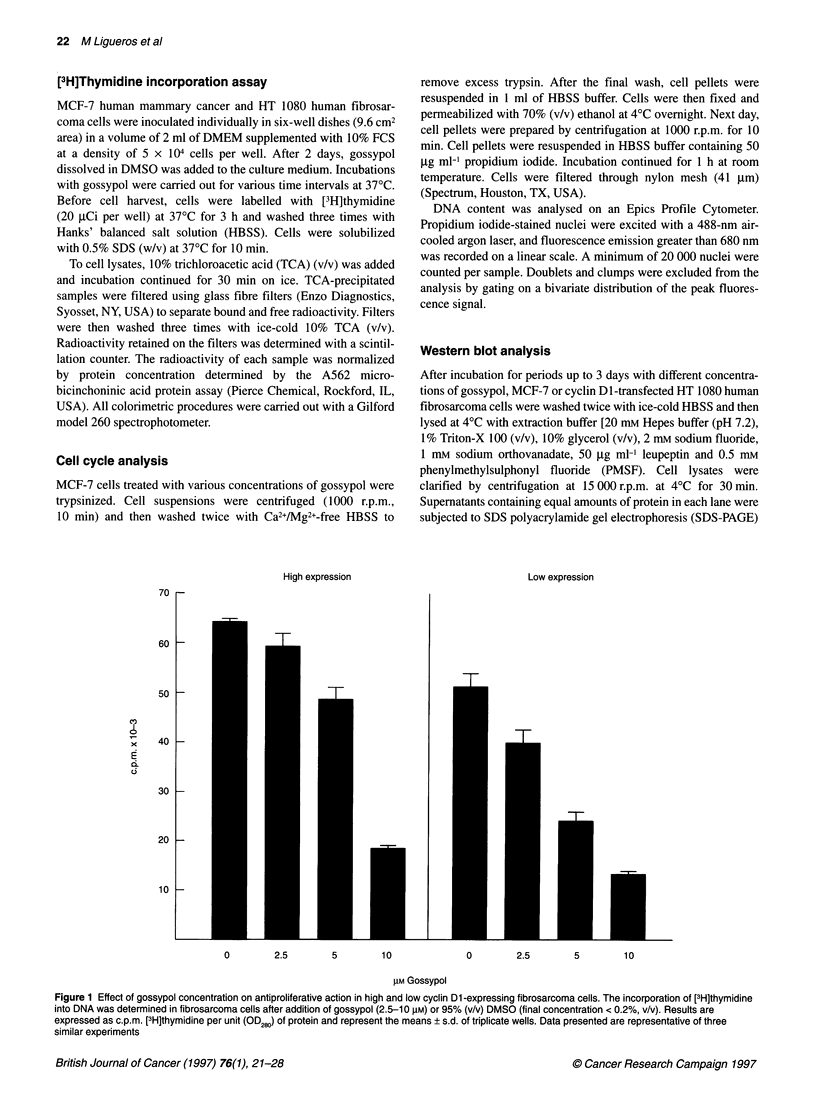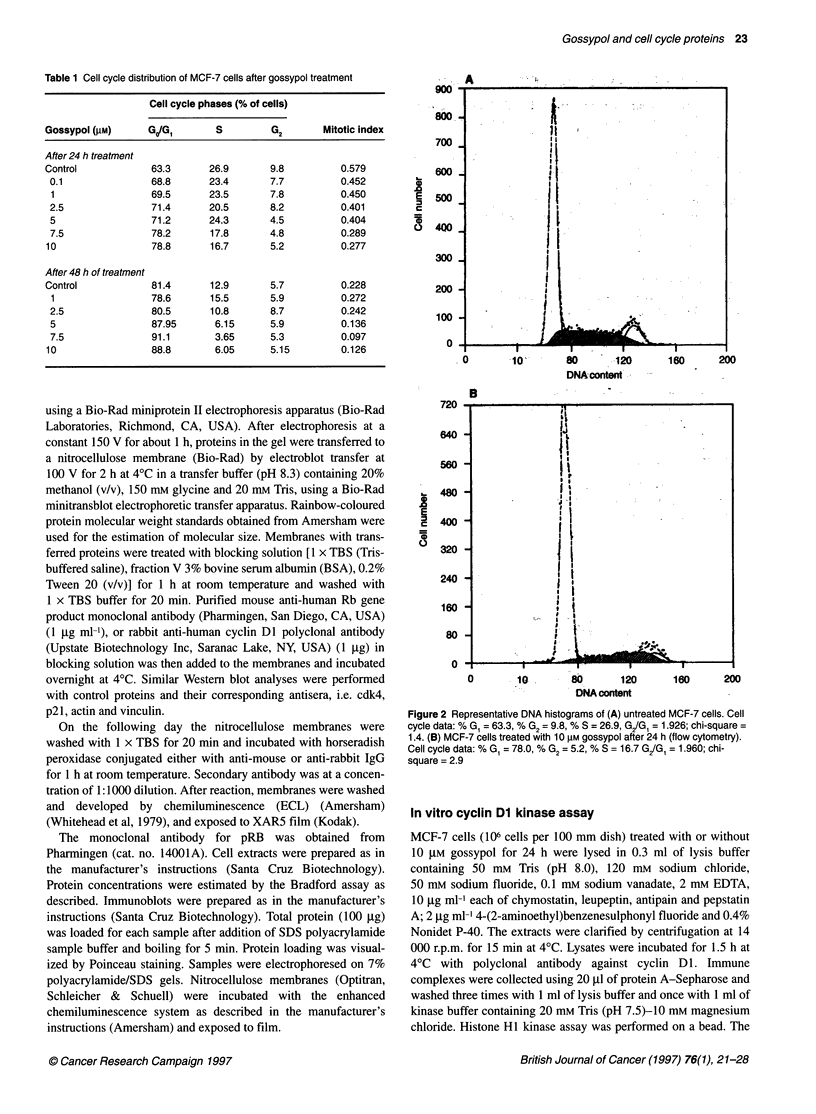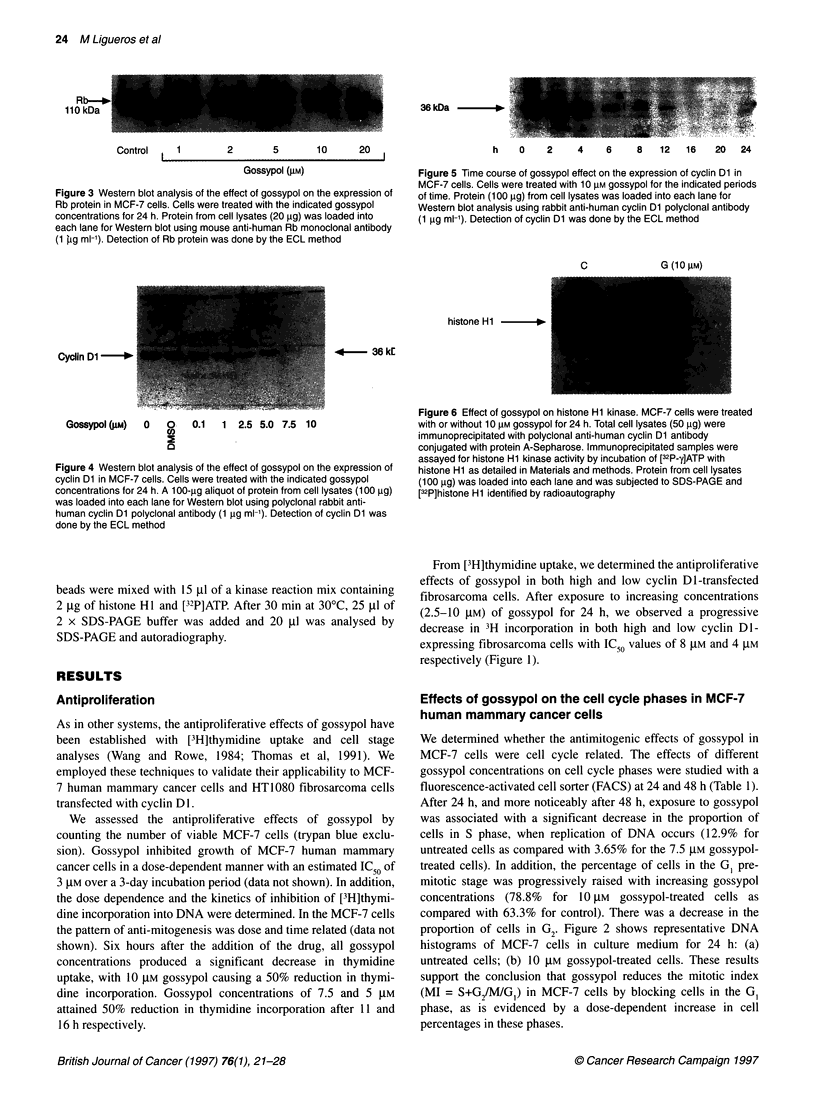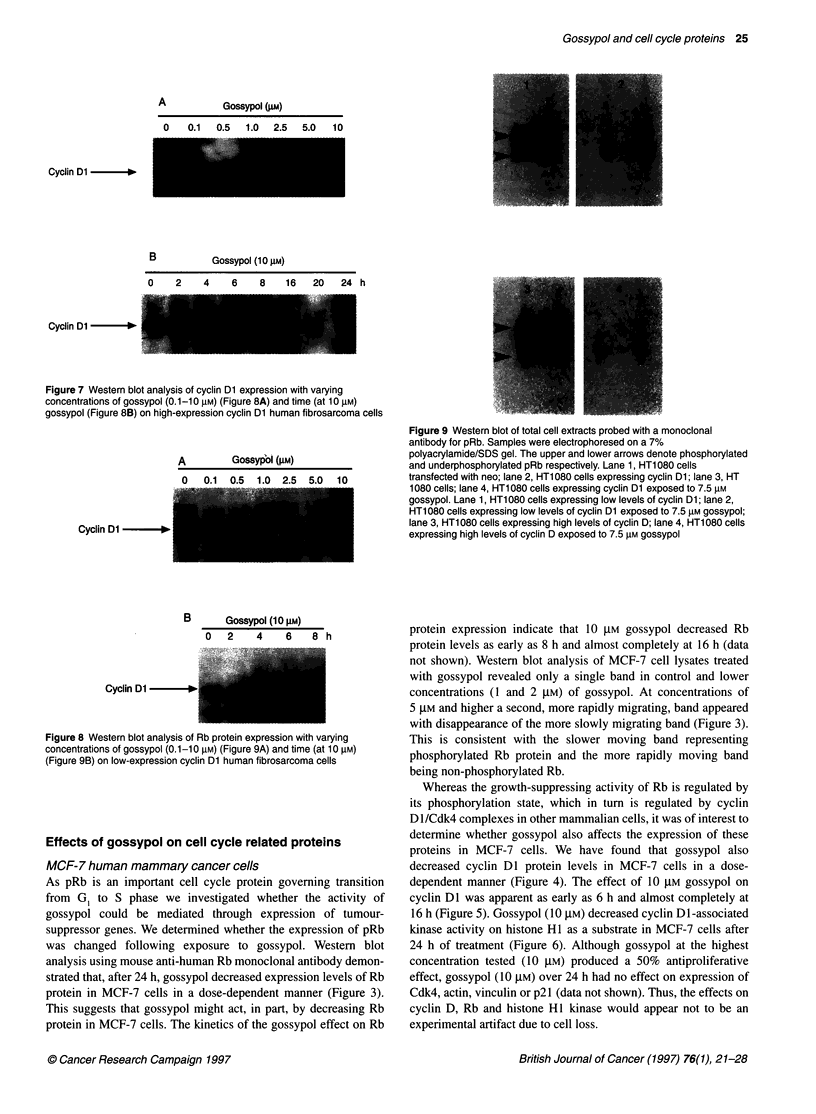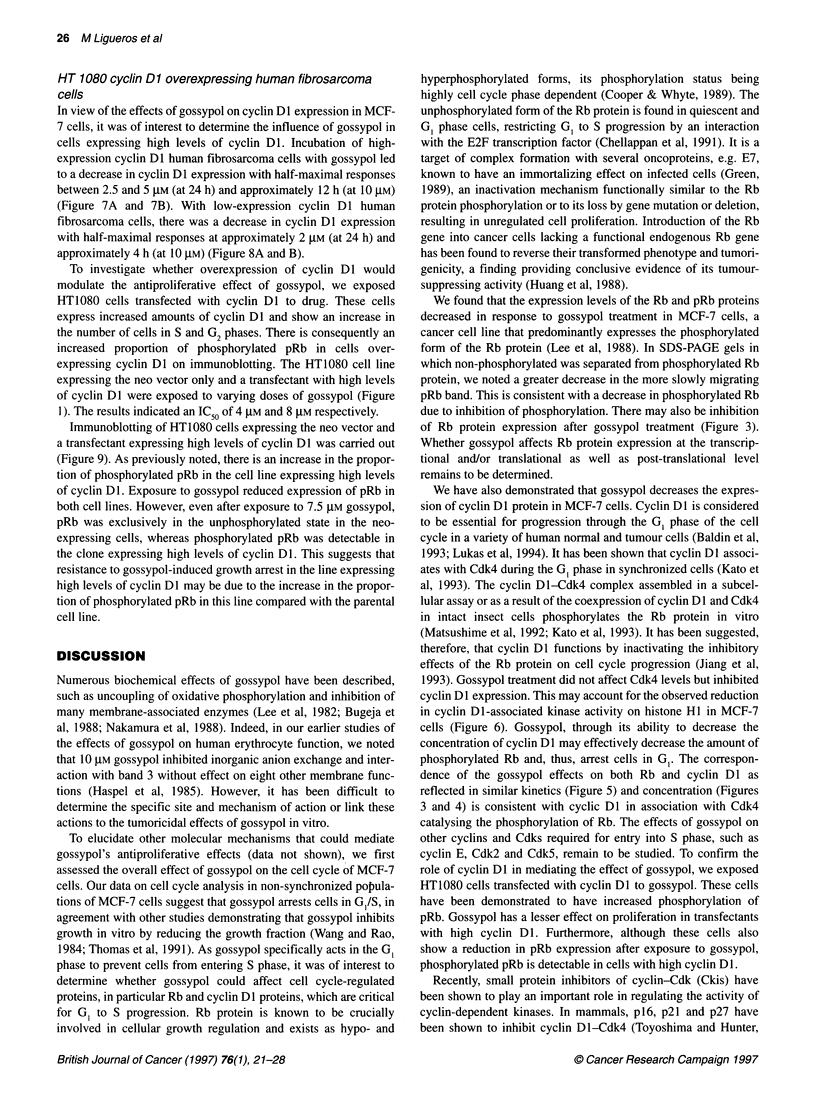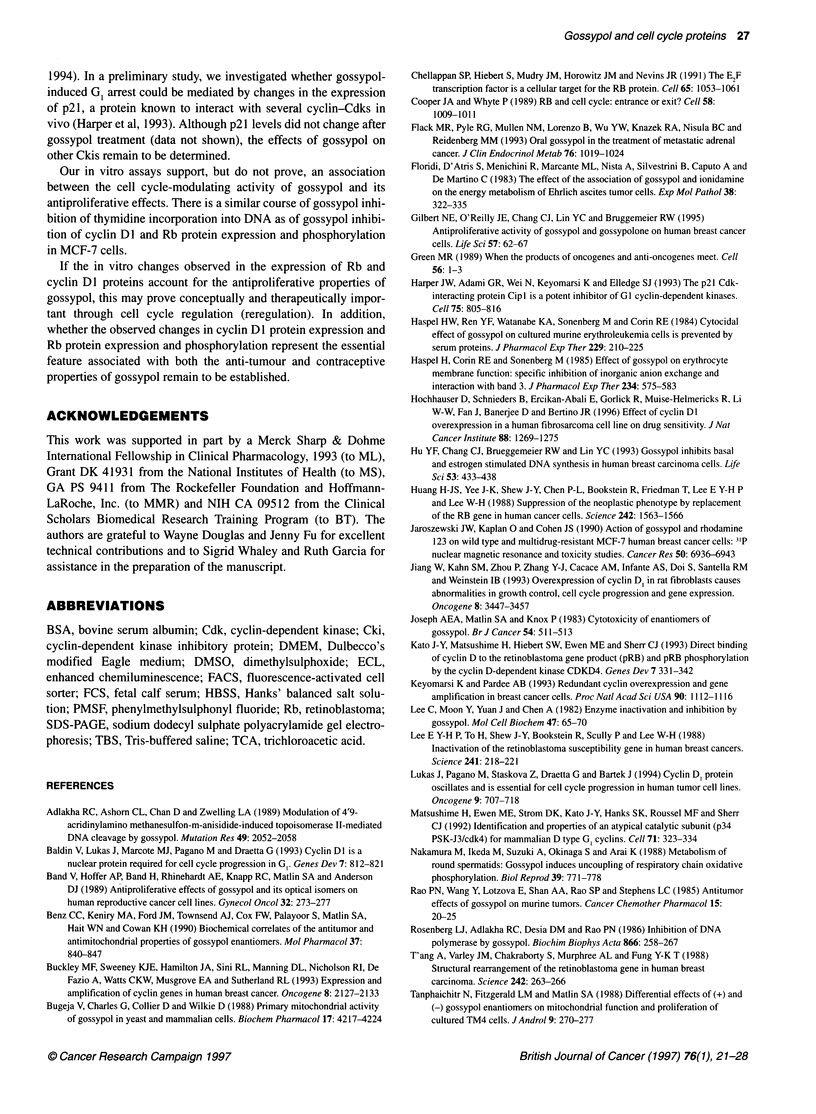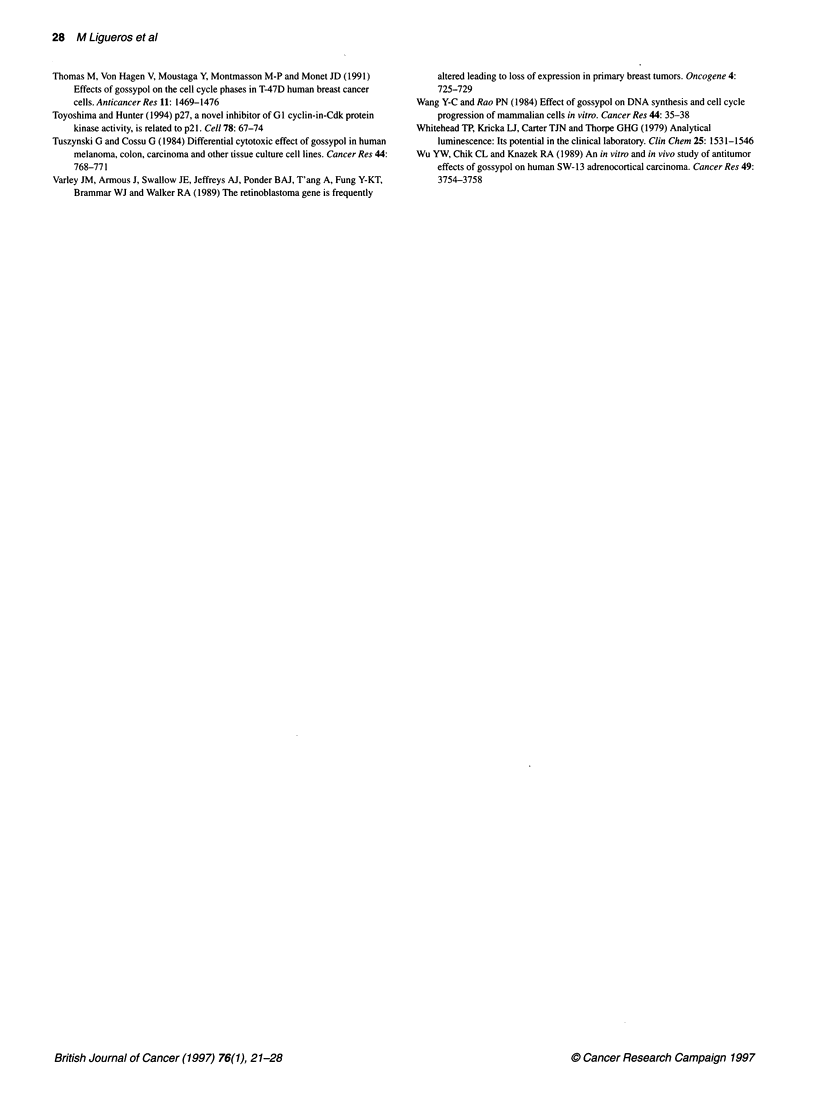# Gossypol inhibition of mitosis, cyclin D1 and Rb protein in human mammary cancer cells and cyclin-D1 transfected human fibrosarcoma cells.

**DOI:** 10.1038/bjc.1997.330

**Published:** 1997

**Authors:** M. Ligueros, D. Jeoung, B. Tang, D. Hochhauser, M. M. Reidenberg, M. Sonenberg

**Affiliations:** Department of Pharmacology, Cornell University Medical College, New York, NY 10021, USA.

## Abstract

**Images:**


British Journal of Cancer (1997) 76(1), 21–28
© 1997 Cancer Research Campaign

### Gossypol inhibition of mitosis, cyclin D1 and Rb protein in human mammary cancer cells and cyclin-D1 transfected human fibrosarcoma cells

M Ligueros[1], D Jeoung[2], B Tang[3], D Hochhauser, MM Reidenberg[1] and M Sonenberg[2]

[1]Departments of Pharmacology and Medicine, Cornell University Medical College, New York, NY 10021; [2]Memorial Sloan-Kettering Cancer Center and Department of Medicine, Cornell University Medical College, New York, NY 10021; USA

**Summary** The antiproliferative effects of gossypol on human MCF-7 mammary cancer cells and cyclin D1-transfected HT-1060 human fibrosarcoma cells were investigated by cell cycle analysis and effects on the cell cycle regulatory proteins Rb and cyclin D1. Flow cytometry of MCF-7 cells at 24 h indicated that 10 μM gossypol inhibited DNA synthesis by producing a $G_1$/S block. Western blot analysis using anti-human Rb antibodies and anti-human cyclin D1 antibodies in MCF-7 cells and high- and low-expression cyclin D1-transfected fibrosarcoma cells indicated that, after 6 h exposure, gossypol decreased the expression levels of these proteins in a dose-dependent manner. Gossypol also decreased the ratio of phosphorylated to unphosphorylated Rb protein in human mammary cancer and fibrosarcoma cell lines. Gossypol (10 μM) treated also decreased cyclin D1-associated kinase activity on histone H1 used as a substrate in MCF-7 cells. These results suggest that gossypol might suppress growth by modulating the expression of cell cycle regulatory proteins Rb and cyclin D1 and the phosphorylation of Rb protein.

**Keywords:** gossypol; mammary cancer; fibrosarcoma; antiproliferation; cell cycle; Rb protein; cyclin D1; phosphorylation of Rb protein

Gossypol, a polyphenolic compound extracted from cotton seeds, has long been recognized as an anti-fertility agent and, more recently, it has been demonstrated to inhibit the growth of various carcinoma cell lines in vitro (Floridi et al, 1983; Joseph et al, 1983; Haspel et al, 1984; Tuszynski and Cossu, 1984; Band et al, 1989; Benz et al, 1990; Jaroszewski et al, 1990) and in vivo (Wu et al, 1989; Rao et al, 1985) including oestrogen-sensitive (MCF-7 and MCF-7 Adr) and -insensitive (MDA-MB-231) human mammary cancer cells (Hu et al, 1993; Gilbert et al, 1995). Clinically, gossypol has been efficacious in the treatment of metastatic adrenal cancer (Flack et al, 1993), holding promise as an anti-tumour agent.

Numerous biochemical studies have been conducted to elucidate the mechanisms by which gossypol exerts its antiproliferative effects (Rosenberg et al, 1986; Adlakha et al, 1989). Data are limited and understanding of gossypol's influence on cell cycle control of DNA synthesis and antimitogenic activity is incomplete (Wang and Rao, 1984; Thomas et al, 1991). We wished to determine whether gossypol could induce changes in the expression of cell cycle regulatory proteins, such as the retinoblastoma (*Rb*) gene product (pRb) and cyclin D1, in human mammary cancer cells, a tumour type associated with mutation of the *Rb* gene (Lee et al, 1988; T'Ang et al, 1988; Varley et al, 1989) as well as overexpression and amplification of cyclin D1 (Buckley et al, 1993; Keyomarsi and Pardee, 1993). In addition, we studied the influence of gossypol on the phosphorylation of Rb protein. For this purpose we used the human mammary cancer cell line MCF-7, which has oestrogen and progesterone receptors, as do certain human cancers in vivo. Additionally, we wished to study gossypol effects on cell cycle phases in MCF-7 cells. Furthermore, to determine the importance of gossypol inhibition on cyclin D1 and Rb protein in its antiproliferative effect, we performed similar studies in human fibrosarcoma cells that overexpress cyclin D1.

Received 9 April 1996
Revised 20 November 1996
Accepted 14 January 1997

Correspondence to: Martin Sonenberg, Memorial Sloan-Kettering Cancer Center, 1275 York Avenue, New York, NY 10021, USA

### MATERIALS AND METHODS

### Cell culture

MCF-7 human mammary cancer cells and HT 1060 human fibrosarcoma cells were obtained from the American Type Culture Collection (Rockville, MD, USA).

The HT 1080 cells were maintained in log-phase growth in RPMI medium (Media Preparation Core Facility, Sloan-Kettering Institute) supplemented with 10% FCS (Sigma, St Louis, MO, USA). Transfection of a cyclin D1-expressing plasmid was carried out as previously described (Hochhauser et al, 1996). Relative expression of cyclin D1 mRNA ratios were 14.4 and 0.48 for the high- and low-expression clones, whereas the comparable cyclin D1 expression ratios were 2.88 and 0.9 as compared with the vector-only-transfected control (Hochhauser et al, 1996).

Racemic gossypol (Sigma) dissolved in dimethylsulphoxide (DMSO) was added to culture medium samples [Dulbecco's modified Eagle medium (DMEM) (Ham's F12/DME, 2:1, v/v) supplemented with 10% fetal calf serum (FCS), 100 μg $ml^{-1}$ streptomycin, 100 U $ml^{-1}$ penicillin and 2 mM glutamine] and incubated at 37°C in a 5% carbon dioxide/95% air atmosphere for 24 h. In order to minimize gossypol oxidation, reduced glutathione was added to the culture medium (2 mM).

### [³H]Thymidine incorporation assay

MCF-7 human mammary cancer and HT 1080 human fibrosarcoma cells were inoculated individually in six-well dishes (9.6 $cm^2$ area) in a volume of 2 ml of DMEM supplemented with 10% FCS at a density of $5 \times 10^4$ cells per well. After 2 days, gossypol dissolved in DMSO was added to the culture medium. Incubations with gossypol were carried out for various time intervals at 37°C. Before cell harvest, cells were labelled with [³H]thymidine (20 μCi per well) at 37°C for 3 h and washed three times with Hanks' balanced salt solution (HBSS). Cells were solubilized with 0.5% SDS (w/v) at 37°C for 10 min.

To cell lysates, 10% trichloroacetic acid (TCA) (v/v) was added and incubation continued for 30 min on ice. TCA-precipitated samples were filtered using glass fibre filters (Enzo Diagnostics, Syosset, NY, USA) to separate bound and free radioactivity. Filters were then washed three times with ice-cold 10% TCA (v/v). Radioactivity retained on the filters was determined with a scintillation counter. The radioactivity of each sample was normalized by protein concentration determined by the A562 micro-bicinchoninic acid protein assay (Pierce Chemical, Rockford, IL, USA). All colorimetric procedures were carried out with a Gilford model 260 spectrophotometer.

### Cell cycle analysis

MCF-7 cells treated with various concentrations of gossypol were trypsinized. Cell suspensions were centrifuged (1000 r.p.m., 10 min) and then washed twice with $Ca^{2+}/Mg^{2+}$-free HBSS to remove excess trypsin. After the final wash, cell pellets were resuspended in 1 ml of HBSS buffer. Cells were then fixed and permeabilized with 70% (v/v) ethanol at 4°C overnight. Next day, cell pellets were prepared by centrifugation at 1000 r.p.m. for 10 min. Cell pellets were resuspended in HBSS buffer containing 50 μg $ml^{-1}$ propidium iodide. Incubation continued for 1 h at room temperature. Cells were filtered through nylon mesh (41 μm) (Spectrum, Houston, TX, USA).

DNA content was analysed on an Epics Profile Cytometer. Propidium iodide-stained nuclei were excited with a 488-nm air-cooled argon laser, and fluorescence emission greater than 680 nm was recorded on a linear scale. A minimum of 20 000 nuclei were counted per sample. Doublets and clumps were excluded from the analysis by gating on a bivariate distribution of the peak fluorescence signal.

### Western blot analysis

After incubation for periods up to 3 days with different concentrations of gossypol, MCF-7 or cyclin D1-transfected HT 1080 human fibrosarcoma cells were washed twice with ice-cold HBSS and then lysed at 4°C with extraction buffer [20 mM Hepes buffer (pH 7.2), 1% Triton-X 100 (v/v), 10% glycerol (v/v), 2 mM sodium fluoride, 1 mM sodium orthovanadate, 50 μg $ml^{-1}$ leupeptin and 0.5 mM phenylmethylsulphonyl fluoride (PMSF). Cell lysates were clarified by centrifugation at 15 000 r.p.m. at 4°C for 30 min. Supernatants containing equal amounts of protein in each lane were subjected to SDS polyacrylamide gel electrophoresis (SDS-PAGE)

**Figure 1** Effect of gossypol concentration on antiproliferative action in high and low cyclin D1-expressing fibrosarcoma cells. The incorporation of [³H]thymidine into DNA was determined in fibrosarcoma cells after addition of gossypol (2.5–10 μM) or 95% (v/v) DMSO (final concentration < 0.2%, v/v). Results are expressed as c.p.m. [³H]thymidine per unit ($OD_{280}$) of protein and represent the means ± s.d. of triplicate wells. Data presented are representative of three similar experiments

 © Cancer Research Campaign 1997

**Table 1** Cell cycle distribution of MCF-7 cells after gossypol treatment

| | Cell cycle phases (% of cells) | | | |
|---|---|---|---|---|
| Gossypol (µM) | $G_0/G_1$ | S | $G_2$ | Mitotic index |
| *After 24 h treatment* | | | | |
| Control | 63.3 | 26.9 | 9.8 | 0.579 |
| 0.1 | 68.8 | 23.4 | 7.7 | 0.452 |
| 1 | 69.5 | 23.5 | 7.8 | 0.450 |
| 2.5 | 71.4 | 20.5 | 8.2 | 0.401 |
| 5 | 71.2 | 24.3 | 4.5 | 0.404 |
| 7.5 | 78.2 | 17.8 | 4.8 | 0.289 |
| 10 | 78.8 | 16.7 | 5.2 | 0.277 |
| *After 48 h of treatment* | | | | |
| Control | 81.4 | 12.9 | 5.7 | 0.228 |
| 1 | 78.6 | 15.5 | 5.9 | 0.272 |
| 2.5 | 80.5 | 10.8 | 8.7 | 0.242 |
| 5 | 87.95 | 6.15 | 5.9 | 0.136 |
| 7.5 | 91.1 | 3.65 | 5.3 | 0.097 |
| 10 | 88.8 | 6.05 | 5.15 | 0.126 |

using a Bio-Rad miniprotein II electrophoresis apparatus (Bio-Rad Laboratories, Richmond, CA, USA). After electrophoresis at a constant 150 V for about 1 h, proteins in the gel were transferred to a nitrocellulose membrane (Bio-Rad) by electroblot transfer at 100 V for 2 h at 4°C in a transfer buffer (pH 8.3) containing 20% methanol (v/v), 150 mM glycine and 20 mM Tris, using a Bio-Rad minitransblot electrophoretic transfer apparatus. Rainbow-coloured protein molecular weight standards obtained from Amersham were used for the estimation of molecular size. Membranes with transferred proteins were treated with blocking solution [1 × TBS (Tris-buffered saline), fraction V 3% bovine serum albumin (BSA), 0.2% Tween 20 (v/v)] for 1 h at room temperature and washed with 1 × TBS buffer for 20 min. Purified mouse anti-human Rb gene product monoclonal antibody (Pharmingen, San Diego, CA, USA) (1 µg $ml^{-1}$), or rabbit anti-human cyclin D1 polyclonal antibody (Upstate Biotechnology Inc, Saranac Lake, NY, USA) (1 µg) in blocking solution was then added to the membranes and incubated overnight at 4°C. Similar Western blot analyses were performed with control proteins and their corresponding antisera, i.e. cdk4, p21, actin and vinculin.

On the following day the nitrocellulose membranes were washed with 1 × TBS for 20 min and incubated with horseradish peroxidase conjugated either with anti-mouse or anti-rabbit IgG for 1 h at room temperature. Secondary antibody was at a concentration of 1:1000 dilution. After reaction, membranes were washed and developed by chemiluminescence (ECL) (Amersham) (Whitehead et al, 1979), and exposed to XAR5 film (Kodak).

The monoclonal antibody for pRB was obtained from Pharmingen (cat. no. 14001A). Cell extracts were prepared as in the manufacturer's instructions (Santa Cruz Biotechnology). Protein concentrations were estimated by the Bradford assay as described. Immunoblots were prepared as in the manufacturer's instructions (Santa Cruz Biotechnology). Total protein (100 µg) was loaded for each sample after addition of SDS polyacrylamide sample buffer and boiling for 5 min. Protein loading was visualized by Poinceau staining. Samples were electrophoresed on 7% polyacrylamide/SDS gels. Nitrocellulose membranes (Optitran, Schleicher & Schuell) were incubated with the enhanced chemiluminescence system as described in the manufacturer's instructions (Amersham) and exposed to film.

**Figure 2** Representative DNA histograms of (**A**) untreated MCF-7 cells. Cell cycle data: % $G_1$ = 63.3, % $G_2$ = 9.8, % S = 26.9, $G_2/G_1$ = 1.926; chi-square = 1.4. (**B**) MCF-7 cells treated with 10 µM gossypol after 24 h (flow cytometry). Cell cycle data: % $G_1$ = 78.0, % $G_2$ = 5.2, % S = 16.7 $G_2/G_1$ = 1.960; chi-square = 2.9

### In vitro cyclin D1 kinase assay

MCF-7 cells ($10^6$ cells per 100 mm dish) treated with or without 10 µM gossypol for 24 h were lysed in 0.3 ml of lysis buffer containing 50 mM Tris (pH 8.0), 120 mM sodium chloride, 50 mM sodium fluoride, 0.1 mM sodium vanadate, 2 mM EDTA, 10 µg $ml^{-1}$ each of chymostatin, leupeptin, antipain and pepstatin A; 2 µg $ml^{-1}$ 4-(2-aminoethyl)benzenesulphonyl fluoride and 0.4% Nonidet P-40. The extracts were clarified by centrifugation at 14 000 r.p.m. for 15 min at 4°C. Lysates were incubated for 1.5 h at 4°C with polyclonal antibody against cyclin D1. Immune complexes were collected using 20 µl of protein A–Sepharose and washed three times with 1 ml of lysis buffer and once with 1 ml of kinase buffer containing 20 mM Tris (pH 7.5)–10 mM magnesium chloride. Histone H1 kinase assay was performed on a bead. The

© Cancer Research Campaign 1997 

**Figure 3** Western blot analysis of the effect of gossypol on the expression of Rb protein in MCF-7 cells. Cells were treated with the indicated gossypol concentrations for 24 h. Protein from cell lysates (20 μg) was loaded into each lane for Western blot using mouse anti-human Rb monoclonal antibody (1 μg ml$^{-1}$). Detection of Rb protein was done by the ECL method

**Figure 5** Time course of gossypol effect on the expression of cyclin D1 in MCF-7 cells. Cells were treated with 10 μM gossypol for the indicated periods of time. Protein (100 μg) from cell lysates was loaded into each lane for Western blot analysis using rabbit anti-human cyclin D1 polyclonal antibody (1 μg ml$^{-1}$). Detection of cyclin D1 was done by the ECL method

**Figure 4** Western blot analysis of the effect of gossypol on the expression of cyclin D1 in MCF-7 cells. Cells were treated with the indicated gossypol concentrations for 24 h. A 100-μg aliquot of protein from cell lysates (100 μg) was loaded into each lane for Western blot using polyclonal rabbit anti-human cyclin D1 polyclonal antibody (1 μg ml$^{-1}$). Detection of cyclin D1 was done by the ECL method

**Figure 6** Effect of gossypol on histone H1 kinase. MCF-7 cells were treated with or without 10 μM gossypol for 24 h. Total cell lysates (50 μg) were immunoprecipitated with polyclonal anti-human cyclin D1 antibody conjugated with protein A-Sepharose. Immunoprecipitated samples were assayed for histone H1 kinase activity by incubation of [$^{32}$P-γ]ATP with histone H1 as detailed in Materials and methods. Protein from cell lysates (100 μg) was loaded into each lane and was subjected to SDS-PAGE and [$^{32}$P]histone H1 identified by radioautography beads were mixed with 15 μl of a kinase reaction mix containing 2 μg of histone H1 and [$^{32}$P]ATP. After 30 min at 30°C, 25 μl of 2 × SDS-PAGE buffer was added and 20 μl was analysed by SDS-PAGE and autoradiography.

### RESULTS

### Antiproliferation

As in other systems, the antiproliferative effects of gossypol have been established with [$^3$H]thymidine uptake and cell stage analyses (Wang and Rowe, 1984; Thomas et al, 1991). We employed these techniques to validate their applicability to MCF-7 human mammary cancer cells and HT1080 fibrosarcoma cells transfected with cyclin D1.

We assessed the antiproliferative effects of gossypol by counting the number of viable MCF-7 cells (trypan blue exclusion). Gossypol inhibited growth of MCF-7 human mammary cancer cells in a dose-dependent manner with an estimated $IC_{50}$ of 3 μM over a 3-day incubation period (data not shown). In addition, the dose dependence and the kinetics of inhibition of [$^3$H]thymidine incorporation into DNA were determined. In the MCF-7 cells the pattern of anti-mitogenesis was dose and time related (data not shown). Six hours after the addition of the drug, all gossypol concentrations produced a significant decrease in thymidine uptake, with 10 μM gossypol causing a 50% reduction in thymidine incorporation. Gossypol concentrations of 7.5 and 5 μM attained 50% reduction in thymidine incorporation after 11 and 16 h respectively.

From [$^3$H]thymidine uptake, we determined the antiproliferative effects of gossypol in both high and low cyclin D1-transfected fibrosarcoma cells. After exposure to increasing concentrations (2.5–10 μM) of gossypol for 24 h, we observed a progressive decrease in $^3$H incorporation in both high and low cyclin D1-expressing fibrosarcoma cells with $IC_{50}$ values of 8 μM and 4 μM respectively (Figure 1).

### Effects of gossypol on the cell cycle phases in MCF-7 human mammary cancer cells

We determined whether the antimitogenic effects of gossypol in MCF-7 cells were cell cycle related. The effects of different gossypol concentrations on cell cycle phases were studied with a fluorescence-activated cell sorter (FACS) at 24 and 48 h (Table 1). After 24 h, and more noticeably after 48 h, exposure to gossypol was associated with a significant decrease in the proportion of cells in S phase, when replication of DNA occurs (12.9% for untreated cells as compared with 3.65% for the 7.5 μM gossypol-treated cells). In addition, the percentage of cells in the $G_1$ premitotic stage was progressively raised with increasing gossypol concentrations (78.8% for 10 μM gossypol-treated cells as compared with 63.3% for control). There was a decrease in the proportion of cells in $G_2$. Figure 2 shows representative DNA histograms of MCF-7 cells in culture medium for 24 h: (a) untreated cells; (b) 10 μM gossypol-treated cells. These results support the conclusion that gossypol reduces the mitotic index (MI = $S+G_2/M/G_1$) in MCF-7 cells by blocking cells in the $G_1$ phase, as is evidenced by a dose-dependent increase in cell percentages in these phases.

© Cancer Research Campaign 1997

**Figure 7** Western blot analysis of cyclin D1 expression with varying concentrations of gossypol (0.1–10 μM) (Figure 8**A**) and time (at 10 μM) gossypol (Figure 8**B**) on high-expression cyclin D1 human fibrosarcoma cells

**Figure 8** Western blot analysis of Rb protein expression with varying concentrations of gossypol (0.1–10 μM) (Figure 9A) and time (at 10 μM) (Figure 9B) on low-expression cyclin D1 human fibrosarcoma cells

### Effects of gossypol on cell cycle related proteins

### *MCF-7 human mammary cancer cells*

As pRb is an important cell cycle protein governing transition from $G_1$ to S phase we investigated whether the activity of gossypol could be mediated through expression of tumour-suppressor genes. We determined whether the expression of pRb was changed following exposure to gossypol. Western blot analysis using mouse anti-human Rb monoclonal antibody demonstrated that, after 24 h, gossypol decreased expression levels of Rb protein in MCF-7 cells in a dose-dependent manner (Figure 3). This suggests that gossypol might act, in part, by decreasing Rb protein in MCF-7 cells. The kinetics of the gossypol effect on Rb

**Figure 9** Western blot of total cell extracts probed with a monoclonal antibody for pRb. Samples were electrophoresed on a 7% polyacrylamide/SDS gel. The upper and lower arrows denote phosphorylated and underphosphorylated pRb respectively. Lane 1, HT1080 cells transfected with neo; lane 2, HT1080 cells expressing cyclin D1; lane 3, HT 1080 cells; lane 4, HT1080 cells expressing cyclin D1 exposed to 7.5 μM gossypol. Lane 1, HT1080 cells expressing low levels of cyclin D1; lane 2, HT1080 cells expressing low levels of cyclin D1 exposed to 7.5 μM gossypol; lane 3, HT1080 cells expressing high levels of cyclin D; lane 4, HT1080 cells expressing high levels of cyclin D exposed to 7.5 μM gossypol protein expression indicate that 10 μM gossypol decreased Rb protein levels as early as 8 h and almost completely at 16 h (data not shown). Western blot analysis of MCF-7 cell lysates treated with gossypol revealed only a single band in control and lower concentrations (1 and 2 μM) of gossypol. At concentrations of 5 μM and higher a second, more rapidly migrating, band appeared with disappearance of the more slowly migrating band (Figure 3). This is consistent with the slower moving band representing phosphorylated Rb protein and the more rapidly moving band being non-phosphorylated Rb.

Whereas the growth-suppressing activity of Rb is regulated by its phosphorylation state, which in turn is regulated by cyclin D1/Cdk4 complexes in other mammalian cells, it was of interest to determine whether gossypol also affects the expression of these proteins in MCF-7 cells. We have found that gossypol also decreased cyclin D1 protein levels in MCF-7 cells in a dose-dependent manner (Figure 4). The effect of 10 μM gossypol on cyclin D1 was apparent as early as 6 h and almost completely at 16 h (Figure 5). Gossypol (10 μM) decreased cyclin D1-associated kinase activity on histone H1 as a substrate in MCF-7 cells after 24 h of treatment (Figure 6). Although gossypol at the highest concentration tested (10 μM) produced a 50% antiproliferative effect, gossypol (10 μM) over 24 h had no effect on expression of Cdk4, actin, vinculin or p21 (data not shown). Thus, the effects on cyclin D, Rb and histone H1 kinase would appear not to be an experimental artifact due to cell loss.

© Cancer Research Campaign 1997

### HT 1080 cyclin D1 overexpressing human fibrosarcoma cells

In view of the effects of gossypol on cyclin D1 expression in MCF-7 cells, it was of interest to determine the influence of gossypol in cells expressing high levels of cyclin D1. Incubation of high-expression cyclin D1 human fibrosarcoma cells with gossypol led to a decrease in cyclin D1 expression with half-maximal responses between 2.5 and 5 μM (at 24 h) and approximately 12 h (at 10 μM) (Figure 7A and 7B). With low-expression cyclin D1 human fibrosarcoma cells, there was a decrease in cyclin D1 expression with half-maximal responses at approximately 2 μM (at 24 h) and approximately 4 h (at 10 μM) (Figure 8A and B).

To investigate whether overexpression of cyclin D1 would modulate the antiproliferative effect of gossypol, we exposed HT1080 cells transfected with cyclin D1 to drug. These cells express increased amounts of cyclin D1 and show an increase in the number of cells in S and $G_2$ phases. There is consequently an increased proportion of phosphorylated pRb in cells over-expressing cyclin D1 on immunoblotting. The HT1080 cell line expressing the neo vector only and a transfectant with high levels of cyclin D1 were exposed to varying doses of gossypol (Figure 1). The results indicated an $IC_{50}$ of 4 μM and 8 μM respectively.

Immunoblotting of HT1080 cells expressing the neo vector and a transfectant expressing high levels of cyclin D1 was carried out (Figure 9). As previously noted, there is an increase in the proportion of phosphorylated pRb in the cell line expressing high levels of cyclin D1. Exposure to gossypol reduced expression of pRb in both cell lines. However, even after exposure to 7.5 μM gossypol, pRb was exclusively in the unphosphorylated state in the neo-expressing cells, whereas phosphorylated pRb was detectable in the clone expressing high levels of cyclin D1. This suggests that resistance to gossypol-induced growth arrest in the line expressing high levels of cyclin D1 may be due to the increase in the proportion of phosphorylated pRb in this line compared with the parental cell line.

### DISCUSSION

Numerous biochemical effects of gossypol have been described, such as uncoupling of oxidative phosphorylation and inhibition of many membrane-associated enzymes (Lee et al, 1982; Bugeja et al, 1988; Nakamura et al, 1988). Indeed, in our earlier studies of the effects of gossypol on human erythrocyte function, we noted that 10 μM gossypol inhibited inorganic anion exchange and interaction with band 3 without effect on eight other membrane functions (Haspel et al, 1985). However, it has been difficult to determine the specific site and mechanism of action or link these actions to the tumoricidal effects of gossypol in vitro.

To elucidate other molecular mechanisms that could mediate gossypol's antiproliferative effects (data not shown), we first assessed the overall effect of gossypol on the cell cycle of MCF-7 cells. Our data on cell cycle analysis in non-synchronized populations of MCF-7 cells suggest that gossypol arrests cells in $G_1$/S, in agreement with other studies demonstrating that gossypol inhibits growth in vitro by reducing the growth fraction (Wang and Rao, 1984; Thomas et al, 1991). As gossypol specifically acts in the $G_1$ phase to prevent cells from entering S phase, it was of interest to determine whether gossypol could affect cell cycle-regulated proteins, in particular Rb and cyclin D1 proteins, which are critical for $G_1$ to S progression. Rb protein is known to be crucially involved in cellular growth regulation and exists as hypo- and hyperphosphorylated forms, its phosphorylation status being highly cell cycle phase dependent (Cooper & Whyte, 1989). The unphosphorylated form of the Rb protein is found in quiescent and $G_1$ phase cells, restricting $G_1$ to S progression by an interaction with the E2F transcription factor (Chellappan et al, 1991). It is a target of complex formation with several oncoproteins, e.g. E7, known to have an immortalizing effect on infected cells (Green, 1989), an inactivation mechanism functionally similar to the Rb protein phosphorylation or to its loss by gene mutation or deletion, resulting in unregulated cell proliferation. Introduction of the Rb gene into cancer cells lacking a functional endogenous Rb gene has been found to reverse their transformed phenotype and tumorigenicity, a finding providing conclusive evidence of its tumour-suppressing activity (Huang et al, 1988).

We found that the expression levels of the Rb and pRb proteins decreased in response to gossypol treatment in MCF-7 cells, a cancer cell line that predominantly expresses the phosphorylated form of the Rb protein (Lee et al, 1988). In SDS-PAGE gels in which non-phosphorylated was separated from phosphorylated Rb protein, we noted a greater decrease in the more slowly migrating pRb band. This is consistent with a decrease in phosphorylated Rb due to inhibition of phosphorylation. There may also be inhibition of Rb protein expression after gossypol treatment (Figure 3). Whether gossypol affects Rb protein expression at the transcriptional and/or translational as well as post-translational level remains to be determined.

We have also demonstrated that gossypol decreases the expression of cyclin D1 protein in MCF-7 cells. Cyclin D1 is considered to be essential for progression through the $G_1$ phase of the cell cycle in a variety of human normal and tumour cells (Baldin et al, 1993; Lukas et al, 1994). It has been shown that cyclin D1 associates with Cdk4 during the $G_1$ phase in synchronized cells (Kato et al, 1993). The cyclin D1–Cdk4 complex assembled in a subcellular assay or as a result of the coexpression of cyclin D1 and Cdk4 in intact insect cells phosphorylates the Rb protein in vitro (Matsushime et al, 1992; Kato et al, 1993). It has been suggested, therefore, that cyclin D1 functions by inactivating the inhibitory effects of the Rb protein on cell cycle progression (Jiang et al, 1993). Gossypol treatment did not affect Cdk4 levels but inhibited cyclin D1 expression. This may account for the observed reduction in cyclin D1-associated kinase activity on histone H1 in MCF-7 cells (Figure 6). Gossypol, through its ability to decrease the concentration of cyclin D1 may effectively decrease the amount of phosphorylated Rb and, thus, arrest cells in $G_1$. The correspondence of the gossypol effects on both Rb and cyclin D1 as reflected in similar kinetics (Figure 5) and concentration (Figures 3 and 4) is consistent with cyclic D1 in association with Cdk4 catalysing the phosphorylation of Rb. The effects of gossypol on other cyclins and Cdks required for entry into S phase, such as cyclin E, Cdk2 and Cdk5, remain to be studied. To confirm the role of cyclin D1 in mediating the effect of gossypol, we exposed HT1080 cells transfected with cyclin D1 to gossypol. These cells have been demonstrated to have increased phosphorylation of pRb. Gossypol has a lesser effect on proliferation in transfectants with high cyclin D1. Furthermore, although these cells also show a reduction in pRb expression after exposure to gossypol, phosphorylated pRb is detectable in cells with high cyclin D1.

Recently, small protein inhibitors of cyclin–Cdk (Ckis) have been shown to play an important role in regulating the activity of cyclin-dependent kinases. In mammals, p16, p21 and p27 have been shown to inhibit cyclin D1–Cdk4 (Toyoshima and Hunter,

 © Cancer Research Campaign 1997

1994). In a preliminary study, we investigated whether gossypol-induced $G_1$ arrest could be mediated by changes in the expression of p21, a protein known to interact with several cyclin–Cdks in vivo (Harper et al, 1993). Although p21 levels did not change after gossypol treatment (data not shown), the effects of gossypol on other Ckis remain to be determined.

Our in vitro assays support, but do not prove, an association between the cell cycle-modulating activity of gossypol and its antiproliferative effects. There is a similar course of gossypol inhibition of thymidine incorporation into DNA as of gossypol inhibition of cyclin D1 and Rb protein expression and phosphorylation in MCF-7 cells.

If the in vitro changes observed in the expression of Rb and cyclin D1 proteins account for the antiproliferative properties of gossypol, this may prove conceptually and therapeutically important through cell cycle regulation (reregulation). In addition, whether the observed changes in cyclin D1 protein expression and Rb protein expression and phosphorylation represent the essential feature associated with both the anti-tumour and contraceptive properties of gossypol remain to be established.

### ACKNOWLEDGEMENTS

This work was supported in part by a Merck Sharp & Dohme International Fellowship in Clinical Pharmacology, 1993 (to ML), Grant DK 41931 from the National Institutes of Health (to MS), GA PS 9411 from The Rockefeller Foundation and Hoffmann-LaRoche, Inc. (to MMR) and NIH CA 09512 from the Clinical Scholars Biomedical Research Training Program (to BT). The authors are grateful to Wayne Douglas and Jenny Fu for excellent technical contributions and to Sigrid Whaley and Ruth Garcia for assistance in the preparation of the manuscript.

### ABBREVIATIONS

BSA, bovine serum albumin; Cdk, cyclin-dependent kinase; Cki, cyclin-dependent kinase inhibitory protein; DMEM, Dulbecco's modified Eagle medium; DMSO, dimethylsulphoxide; ECL, enhanced chemiluminescence; FACS, fluorescence-activated cell sorter; FCS, fetal calf serum; HBSS, Hanks' balanced salt solution; PMSF, phenylmethylsulphonyl fluoride; Rb, retinoblastoma; SDS-PAGE, sodium dodecyl sulphate polyacrylamide gel electrophoresis; TBS, Tris-buffered saline; TCA, trichloroacetic acid.

### REFERENCES

Adlakha RC, Ashorn CL, Chan D and Zwelling LA (1989) Modulation of 4′9-acridinylamino methanesulfon-m-anisidide-induced topoisomerase II-mediated DNA cleavage by gossypol. *Mutation Res* **49**: 2052–2058

Baldin V, Lukas J, Marcote MJ, Pagano M and Draetta G (1993) Cyclin D1 is a nuclear protein required for cell cycle progression in $G_1$. *Genes Dev* **7**: 812–821

Band V, Hoffer AP, Band H, Rhinehardt AE, Knapp RC, Matlin SA and Anderson DJ (1989) Antiproliferative effects of gossypol and its optical isomers on human reproductive cancer cell lines. *Gynecol Oncol* **32**: 273–277

Benz CC, Keniry MA, Ford JM, Townsend AJ, Cox FW, Palayoor S, Matlin SA, Hait WN and Cowan KH (1990) Biochemical correlates of the antitumor and antimitochondrial properties of gossypol enantiomers. *Mol Pharmacol* **37**: 840–847

Buckley MF, Sweeney KJE, Hamilton JA, Sini RL, Manning DL, Nicholson RI, De Fazio A, Watts CKW, Musgrove EA and Sutherland RL (1993) Expression and amplification of cyclin genes in human breast cancer. *Oncogene* **8**: 2127–2133

Bugeja V, Charles G, Collier D and Wilkie D (1988) Primary mitochondrial activity of gossypol in yeast and mammalian cells. *Biochem Pharmacol* **17**: 4217–4224

Chellappan SP, Hiebert S, Mudry JM, Horowitz JM and Nevins JR (1991) The $E_2F$ transcription factor is a cellular target for the RB protein. *Cell* **65**: 1053–1061

Cooper JA and Whyte P (1989) RB and cell cycle: entrance or exit? *Cell* **58**: 1009–1011

Flack MR, Pyle RG, Mullen NM, Lorenzo B, Wu YW, Knazek RA, Nisula BC and Reidenberg MM (1993) Oral gossypol in the treatment of metastatic adrenal cancer. *J Clin Endocrinol Metab* **76**: 1019–1024

Floridi, D'Atris S, Menichini R, Marcante ML, Nista A, Silvestrini B, Caputo A and De Martino C (1983) The effect of the association of gossypol and ionidamine on the energy metabolism of Ehrlich ascites tumor cells. *Exp Mol Pathol* **38**: 322–335

Gilbert NE, O'Reilly JE, Chang CJ, Lin YC and Bruggemeier RW (1995) Antiproliferative activity of gossypol and gossypolone on human breast cancer cells. *Life Sci* **57**: 62–67

Green MR (1989) When the products of oncogenes and anti-oncogenes meet. *Cell* **56**: 1–3

Harper JW, Adami GR, Wei N, Keyomarsi K and Elledge SJ (1993) The p21 Cdk-interacting protein Cip1 is a potent inhibitor of G1 cyclin-dependent kinases. *Cell* **75**: 805–816

Haspel HW, Ren YF, Watanabe KA, Sonenberg M and Corin RE (1984) Cytocidal effect of gossypol on cultured murine erythroleukemia cells is prevented by serum proteins. *J Pharmacol Exp Ther* **229**: 210–225

Haspel H, Corin RE and Sonenberg M (1985) Effect of gossypol on erythrocyte membrane function: specific inhibition of inorganic anion exchange and interaction with band 3. *J Pharmacol Exp Ther* **234**: 575–583

Hochhauser D, Schnieders B, Ercikan-Abali E, Gorlick R, Muise-Helmericks R, Li W-W, Fan J, Banerjee D and Bertino JR (1996) Effect of cyclin D1 overexpression in a human fibrosarcoma cell line on drug sensitivity. *J Nat Cancer Institute* **88**: 1269–1275

Hu YF, Chang CJ, Brueggemeier RW and Lin YC (1993) Gossypol inhibits basal and estrogen stimulated DNA synthesis in human breast carcinoma cells. *Life Sci* **53**: 433–438

Huang H-JS, Yee J-K, Shew J-Y, Chen P-L, Bookstein R, Friedman T, Lee E Y-H P and Lee W-H (1988) Suppression of the neoplastic phenotype by replacement of the RB gene in human cancer cells. *Science* **242**: 1563–1566

Jaroszewski JW, Kaplan O and Cohen JS (1990) Action of gossypol and rhodamine 123 on wild type and multidrug-resistant MCF-7 human breast cancer cells: $^{31}P$ nuclear magnetic resonance and toxicity studies. *Cancer Res* **50**: 6936–6943

Jiang W, Kahn SM, Zhou P, Zhang Y-J, Cacace AM, Infante AS, Doi S, Santella RM and Weinstein IB (1993) Overexpression of cyclin $D_1$ in rat fibroblasts causes abnormalities in growth control, cell cycle progression and gene expression. *Oncogene* **8**: 3447–3457

Joseph AEA, Matlin SA and Knox P (1983) Cytotoxicity of enantiomers of gossypol. *Br J Cancer* **54**: 511–513

Kato J-Y, Matsushime H, Hiebert SW, Ewen ME and Sherr CJ (1993) Direct binding of cyclin D to the retinoblastoma gene product (pRB) and pRB phosphorylation by the cyclin D-dependent kinase CDKD4. *Genes Dev* **7** 331–342

Keyomarsi K and Pardee AB (1993) Redundant cyclin overexpression and gene amplification in breast cancer cells. *Proc Natl Acad Sci USA* **90**: 1112–1116

Lee C, Moon Y, Yuan J and Chen A (1982) Enzyme inactivation and inhibition by gossypol. *Mol Cell Biochem* **47**: 65–70

Lee E Y-H P, To H, Shew J-Y, Bookstein R, Scully P and Lee W-H (1988) Inactivation of the retinoblastoma susceptibility gene in human breast cancers. *Science* **241**: 218–221

Lukas J, Pagano M, Staskova Z, Draetta G and Bartek J (1994) Cyclin $D_1$ protein oscillates and is essential for cell cycle progression in human tumor cell lines. *Oncogene* **9**: 707–718

Matsushime H, Ewen ME, Strom DK, Kato J-Y, Hanks SK, Roussel MF and Sherr CJ (1992) Identification and properties of an atypical catalytic subunit (p34 PSK-J3/cdk4) for mammalian D type $G_1$ cyclins. *Cell* **71**: 323–334

Nakamura M, Ikeda M, Suzuki A, Okinaga S and Arai K (1988) Metabolism of round spermatids: Gossypol induces uncoupling of respiratory chain oxidative phosphorylation. *Biol Reprod* **39**: 771–778

Rao PN, Wang Y, Lotzova E, Shan AA, Rao SP and Stephens LC (1985) Antitumor effects of gossypol on murine tumors. *Cancer Chemother Pharmacol* **15**: 20–25

Rosenberg LJ, Adlakha RC, Desia DM and Rao PN (1986) Inhibition of DNA polymerase by gossypol. *Biochim Biophys Acta* **866**: 258–267

T'ang A, Varley JM, Chakraborty S, Murphree AL and Fung Y-K T (1988) Structural rearrangement of the retinoblastoma gene in human breast carcinoma. *Science* **242**: 263–266

Tanphaichitr N, Fitzgerald LM and Matlin SA (1988) Differential effects of (+) and (–) gossypol enantiomers on mitochondrial function and proliferation of cultured TM4 cells. *J Androl* **9**: 270–277

Thomas M, Von Hagen V, Moustaga Y, Montmasson M-P and Monet JD (1991) Effects of gossypol on the cell cycle phases in T-47D human breast cancer cells. *Anticancer Res* **11**: 1469–1476

Toyoshima and Hunter (1994) p27, a novel inhibitor of G1 cyclin-in-Cdk protein kinase activity, is related to p21. *Cell* **78**: 67–74

Tuszynski G and Cossu G (1984) Differential cytotoxic effect of gossypol in human melanoma, colon, carcinoma and other tissue culture cell lines. *Cancer Res* **44**: 768–771

Varley JM, Armous J, Swallow JE, Jeffreys AJ, Ponder BAJ, T'ang A, Fung Y-KT, Brammar WJ and Walker RA (1989) The retinoblastoma gene is frequently altered leading to loss of expression in primary breast tumors. *Oncogene* **4**: 725–729

Wang Y-C and *Rao* PN (1984) Effect of gossypol on DNA synthesis and cell cycle progression of mammalian cells *in vitro*. *Cancer Res* **44**: 35–38

Whitehead TP, Kricka LJ, Carter TJN and Thorpe GHG (1979) Analytical luminescence: Its potential in the clinical laboratory. *Clin Chem* **25**: 1531–1546

Wu YW, Chik CL and Knazek RA (1989) An *in vitro* and *in vivo* study of antitumor effects of gossypol on human SW-13 adrenocortical carcinoma. *Cancer Res* **49**: 3754–3758

© Cancer Research Campaign 1997